# P-1405. Clinical Outcomes and Risk Factors of Favorable and Unfavorable Treatment Outcomes of Drug Resistant-Tuberculosis Individuals on Treatment Using BPaLM/BPaL Regimen Enrolled at San Lazaro Hospital

**DOI:** 10.1093/ofid/ofaf695.1592

**Published:** 2026-01-11

**Authors:** Rodrigo Jon Eva, Rugaiya Calapis, Edna M Edrada, Rontgene M Solante

**Affiliations:** San Lazaro Hospital, Manila, National Capital Region, Philippines; San Lazaro Hospital, Manila, National Capital Region, Philippines; San Lazaro Hospital, Manila, National Capital Region, Philippines; San Lazaro Hospital, Manila, National Capital Region, Philippines

## Abstract

**Background:**

World Health Organization proposed a new drug regimen using BPaLM/BPaL for drug resistant tuberculosis shortening the duration for 6 months and was adopted locally last December 2023. Evidence of its effectivity and treatment outcomes in a developing country with high TB burden is limited, hence a need for this study is important.

**Table 1.**
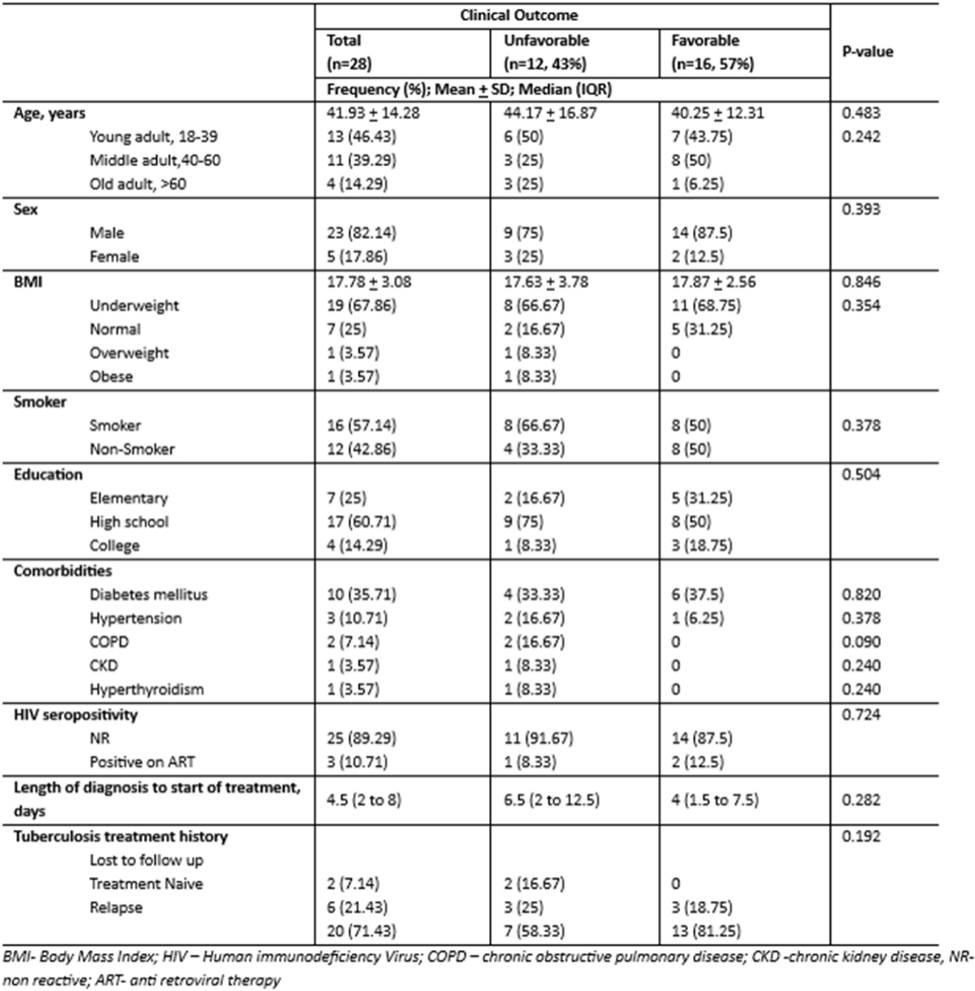
Baseline characteristics of patients with MDR/TT-TB (n = 28) This table shows the baseline characteristics of patients in correlation with favorable and unfavorable outcomes

**Table 2.**
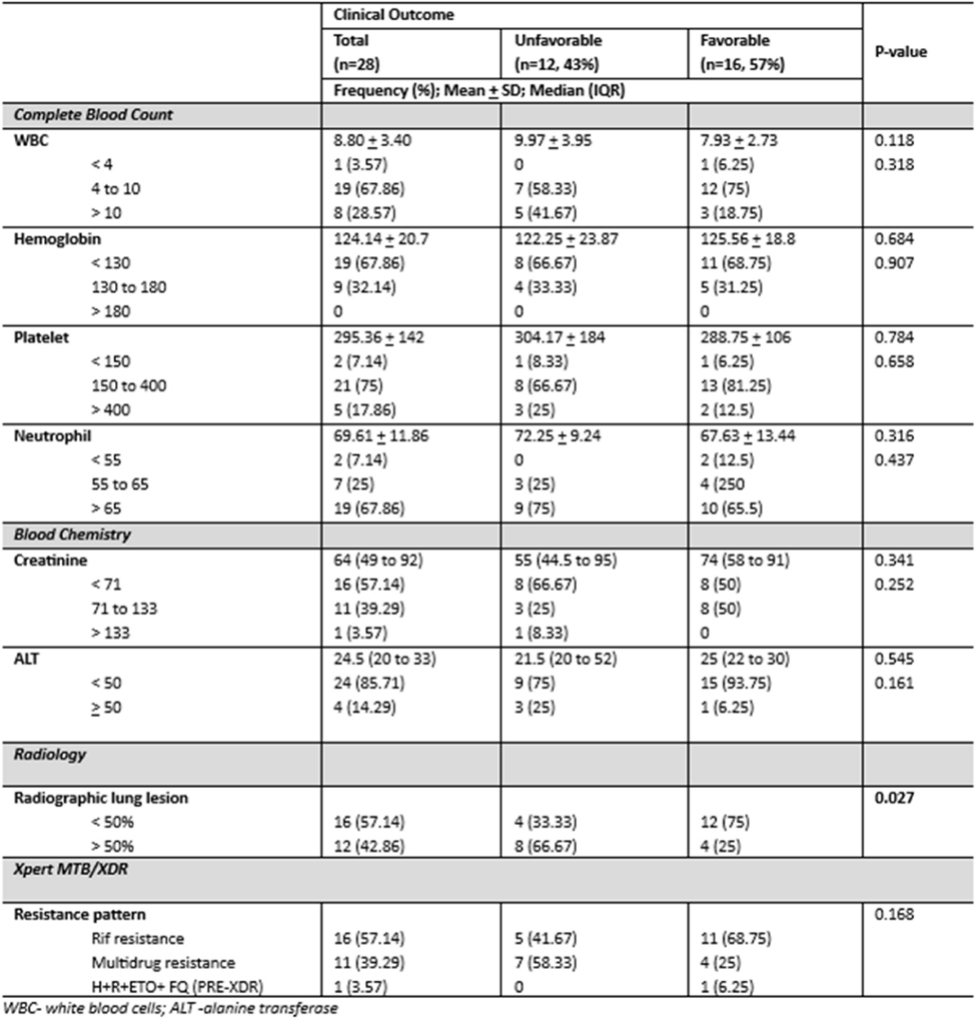
Diagnostic Tests of Patients with MDR/RR-TB (n = 28) This table shows the baseline diagnostic tests of patients in correlation with favorable and unfavorable outcomes

**Methods:**

This is a descriptive, observational study with retrospective chart review of all treated adult patients using BPaLM/BPaL in San Lazaro Hospital, Philippines between February 1, 2024 to February 28,2025 with outcomes.

**Figure 1.**
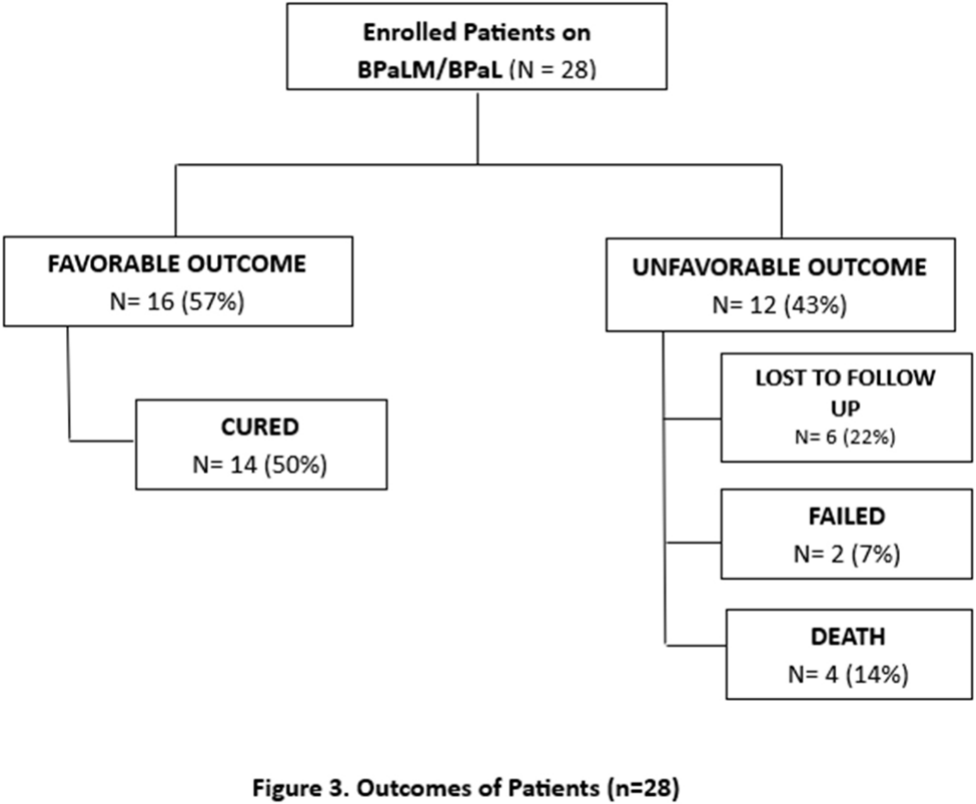
Outcomes of Patients (n=28) This figure shows the treatment outcome of subjects in the study

**Table 3.**
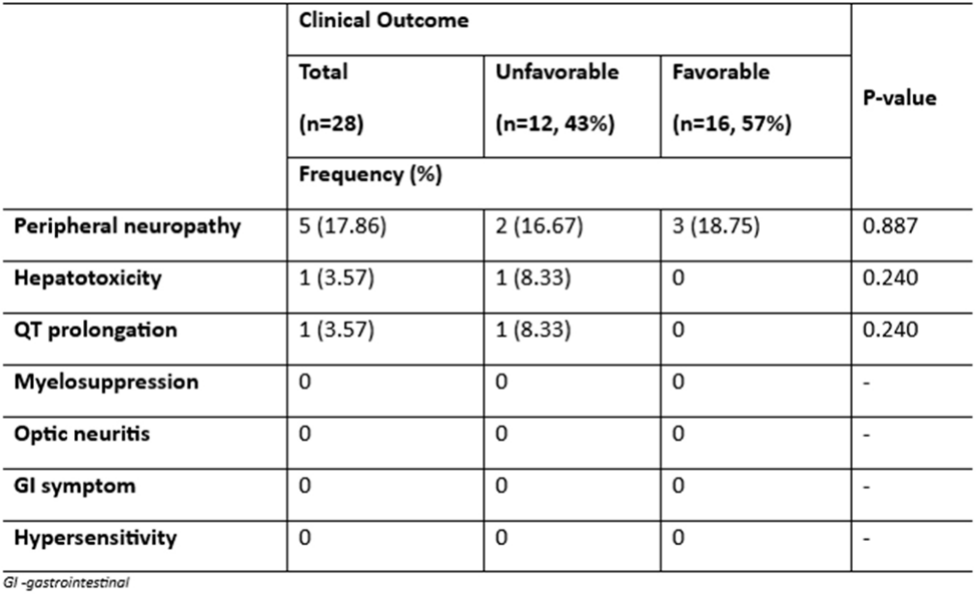
Adverse Events of Patients with MDR/RR-TB (n =28) Table shows the adverse events noted during treatment period

**Results:**

A total of 28 patients were included in the study, average age was 41 years, predominantly male and majority were underweight. Majority of the study population had previous history of tuberculosis treatment (71.43%), 7.14% were lost to follow up and 21.4% were treatment naïve. Demographic characteristics and laboratory analyses did not significantly differ between the two outcome groups (p-values > 0.05). Only radiographic lung lesion extending > 50% had noted significant proportion of patients with unfavorable outcomes. Sixteen patients (57%) had favorable outcomes and completed 6 months of treatment. The longest sputum conversion to a negative culture was 8 weeks. Twelve (43%) had unfavorable outcomes, 6 of the subjects were lost to follow up. Four subjects died due to causes not directly attributable to treatment regimen (myocardial infarction, pneumonia and pneumothorax). Two had pharmacologic treatment failure due to drug induced liver injury and qt prolongation. Adverse events were uncommon, and peripheral neuropathy has highest reported cases at 17.86% a total of 7 (25%) had adverse events during treatment.

**Conclusion:**

Study shows that > 50% radiographic lung lesion are 6 times more likely to have unfavorable outcome and closer monitoring is needed. Favorable outcomes were affected by multiple lost to follow up and death that is not directly attributable to regimen. Looking closely, cured patients had conversion rate of sputum TB culture at 93% on 1st month, 97% on 2nd month and 100% thereafter within 6 months with no noted TB culture reversion, indicating effective and fast killing of bacilli. Adverse events were minimal and safety profile of the regimen was generally acceptable. Importance of adherence must be emphasized.

**Disclosures:**

All Authors: No reported disclosures

